# Dielectric and magnetic characteristics of Ca_1−*x*_Mn_*x*_MoO_4_ (0 ≤ *x* ≤ 0.15) nanomaterials

**DOI:** 10.1007/s11051-018-4450-9

**Published:** 2018-12-31

**Authors:** T. Groń, M. Karolewicz, E. Tomaszewicz, M. Guzik, M. Oboz, B. Sawicki, H. Duda, Z. Kukuła

**Affiliations:** 1 Institute of Physics, University of Silesia, Katowice, Poland; 2 Faculty of Chemical Technology and Engineering, Department of Inorganic and Analytical Chemistry, West Pomeranian University of Technology in Szczecin, Al. Piastów 42, 71-065 Szczecin, Poland; 3 Faculty of Chemistry, University of Wrocław, ul.F. Joliot-Curie 14, 50-383 Wrocław, Poland

**Keywords:** Scheelite-type structure, Mn^2+^-doped nanomaterials, Combustion synthesis, Electrical properties, Magnetic properties

## Abstract

Scheelite-type Ca_1−*x*_Mn_*x*_MoO_4_ (*x* = 0.0, 0.01, 0.05, 0.10 and 0.15) nanomaterials were successfully synthesized via a combustion route. Dielectric studies showed a weak *n*-type electrical conductivity characteristic for insulators and low relative permittivity (*ε*_r_ < 15) decreasing with increasing Mn^2+^ content. CaMoO_4_ and Mn^2+^-doped nanomaterials are chemically compatible with Al and Ag electrodes and promising for low-temperature co-fired ceramic applications. Magnetic studies showed, at room-temperature diamagnetism for pure CaMoO_4_, the balance between diamagnetism and paramagnetism for Ca_1−*x*_Mn_*x*_MoO_4_ (*x* = 0.01) and paramagnetic behaviour when 0.05 ≤ *x* ≤ 0.15 as well as the short-range antiferromagnetic interactions growing in strength as Mn^2+^ content increases. The Landé factor fitting procedure showed a spin-only contribution to the magnetic moment. CaMoO_4_ matrix unexpectedly revealed the residual paramagnetism at low temperatures derived probably from the molybdenum ions having unpaired 4*d* electrons as well as a paramagnetic-diamagnetic transition at 70 K.

## Introduction

Metal molybdates form a wide and important class of inorganic materials that have a high application potential in various fields such as scintillator detectors, phosphors and electro-optic applications (Zhang et al. 2015; Bavykina et al. 2009; Zhou et al. 2011; Danevich et al. 2014; Belogurov et al. 2005; Mikhailik et al. 2007; Korzhik et al. 2008; Lei and Yan 2008; Shivakumara et al. 2015). Divalent metal molybdates with relatively large cations such as Ca^2+^, Sr^2+^, Ba^2+^ and Pb^2+^ (ionic radius above 99 pm) exist in scheelite-type structure (tetragonal symmetry, space group *I4*_*1*_*/a*; *Z* = 4) where molybdenum ions adopt tetrahedral coordination, while divalent metal ions represent an eight-coordinated position (Sczancoski et al. 2008; Sczancoski et al. 2009; Ghosh et al. 2015). Generally, metal molybdates are obtained by conventional solid-state reaction method (Pawlikowska et al. 2017; Piątkowska and Tomaszewicz 2016; Tomaszewicz et al. 2016). However, powders obtained in this way are characterized by large and irregular grains. Furthermore, molybdenum oxide has a tendency to vaporize at high temperatures and inhomogeneous compounds might be easily formed. These problems can be avoided by applying combustion synthesis, wet chemical route, Pechini and other methods (Pawlikowska et al. 2017; Thongtem et al. 2010; Marques et al. 2010; Gong et al. 2006; Chen et al. 2006; Piątkowska et al. 2017).

Rapid growth of wireless communication within last years has caused increasing expectations for new microwave dielectric materials. Such new dielectric ceramics can be applied as band gap filters, antenna switches and dielectric resonators in mobile and satellite communications, intelligent transport systems, voltage-controlled oscillators and duplexers. These systems operating at high frequencies require new materials with relatively low dielectric permittivity (*ε*_r_), high-quality factor (Qxf) and stable and near-to-zero-temperature coefficient of resonant frequency (*τ*_f_), as well as co-fired with internal metal electrodes, i.e. Al, Ag or Cu (Kim et al. 2006; Vidya et al. 2013; Xi et al. 2014; Choi et al. 2007). In particular, a low value of *ε*_r_ is important because a signal propagation velocity is a function of dielectric permittivity. Low-temperature co-fired ceramic (LTCC) technology allows to combine thin layers of dielectric ceramics and conducting electrodes to produce multilayer modules. Pure scheelites such as CaWO_4_, BaWO_4_, PbMoO_4_ and AMoO_4_ (A = Ca, Sr and Ba) were investigated and reported with good microwave dielectric properties (Kim et al. 2006; Vidya et al. 2013; Xi et al. 2014; Choi et al. 2007). The microwave dielectric properties for microcrystalline calcium molybdate (CaMoO_4_) sintered at 1373 K were found to be the dielectric permittivity as 10.79, the high-quality factor as 89,700 GHz and the temperature coefficient of resonant frequency as − 57 ppm/°C (Choi et al. 2007). Its chemical compatibility with some internal electrode such as Al, Ag or Cu has not been investigated so far.

In the present work, CaMoO_4_ and Ca_1−*x*_Mn_*x*_MoO_4_ (*x* = 0.01, 0.05, 0.10 and 0.15) nanomaterials were successfully synthesized via citrate-nitrate combustion route. Low-sintering temperature behaviour, phase composition, microstructure, some dielectric properties and chemical compatibility with metallic aluminium and silver of these nanomaterials were investigated to meet the requirement of microwave devices. Furthermore, magnetic properties of pure matrix and Mn^2+^-doped nanomaterials were investigated. The Landé factor estimated from the Curie constant was used to interpret the magnetic contributions to the magnetic interactions.

## Experimental

### Materials

The following precursors were used in a synthesis: manganese oxide (MnO; Fluka), calcium carbonate (CaCO_3_; Alfa Aesar) and ammonium molybdate ((NH_4_)_6_Mo_7_O_24_·1.3586H_2_O; Alfa Aesar). Citric acid monohydrate (C_6_H_8_O_7_·H_2_O; Alfa Aesar) was used as a fuel. All the solid reagents used were of analytical purity and without further purification. In addition, nitric acid (~ 30%) and ammonia (~ 25%) were used as the aid reactants during a combustion synthesis. For analysis of a chemical compatibility, Al and Ag powders (both metals with purity of 99.9%, 4–7 μm; Alfa Aesar) were applied.

### Combustion synthesis of Mn^2+^-doped nanomaterials

Nanocrystalline samples of pure CaMoO_4_ and a solid solution described by the formula of Ca_1−*x*_Mn_*x*_MoO_4_ (*x* = 0.01, 0.05, 0.10 and 0.15) were successfully obtained via citrate-nitrate combustion route. In the first step, an adequate amount of MnO (0.0353 g; 0.498 mmol when *x* = 0.05) and CaCO_3_ (0.9471 g; 9.463 mmol when *x* = 0.05) was dissolved in hot aqueous solution of nitric acid. Then, citric acid (5.2337 g; 24.906 mmol when *x* = 0.05) and deionized water were added to the solution containing Mn^2+^ and Ca^2+^ ions. The pH of as-obtained solution (solution A) was adjusted to the value of ~ 5 with an ammonia solution. Precursor of Mo (1.6911 g; 1.423 mmol when *x* = 0.05) was dissolved in hot and deionized water (solution B). Next, the both solutions were mixed together and gently heated to completely evaporate water. In the next step, obtained pink gel was heated carefully at 473 K. During the combustion process, the gel has burned out with a rapid evolution of a large quantity of fume, yielding voluminous powder. In the last step, the as-prepared nanomaterials were heated at 723 K for 2 h in air to obtain the final white or light grey product (~ 2 g; ~ 9.962 mmol when *x* = 0.05). To investigate a chemical compatibility of CaMoO_4_ and Mn^2+^-doped nanomaterials with aluminum and silver powders, 30 mass% Al as well as 30 mass% Ag were mixed with CaMoO_4_ and Ca_1−*x*_Mn_*x*_MoO_4_ (*x* = 0.05) nanomaterials and co-fired at 873 K for 4 h.

### Analysis and material characterization

Powder X-ray diffraction patterns of CaMoO_4_ and manganese-doped nanomaterials were collected within the 10–100° 2*θ* range with the scanning step of 0.013° on an Empyrean II diffractometer (PANalytical) using CuK_α1,2_ radiation (*λ* = 0.15418 nm). XRD patterns were analysed by *HighScore Plus 4.0* software. Lattice constants were calculated using the least-squares refinement procedure and *Powder* software (Taupin 1968; Taupin 1973).. The morphology and grain size of Mn^2+^-doped nanomaterials were observed by scanning electron microscopy (SEM) using a Hitachi S-3400N equipped with an energy-dispersive X-ray spectroscopy (EDS) detector. Thermo Scientific UltraDry was used. The powders were coated with a thin gold alloy layer to facilitate conductivity. Transmission electron microscopy (TEM) measurements were carried out for nanopowdered samples on a HRTEM (FEI Titan^3^™ G2 60-300; FEI, Hillsboro, USA). The electrical conductivity (*σ*(*T*)) of the nanoceramics under study was measured within the temperature range of 76–400 K by the DC method using a Keithley 6517B Electrometer/High Resistance Meter. The thermoelectric power (*S*(*T*)) was measured within the temperature range of 300–600 K with the help of a Seebeck Effect Measurement System (MMR Technologies, Inc., USA). Broadband dielectric spectroscopy measurements were carried out using pellets, polished and sputtered with (~ 80 nm) Ag electrodes within the frequency range of 5·10^2^–1·10^6^ Hz using a Hioki 3532-50 LCR HiTester (TEquipment.NET, LLC Company, USA) and within the temperature range of 76–400 K. For the electrical measurements, the powder samples were compacted in a disc form (10 mm in diameter and 1–2 mm in thickness) using a pressure of 1.5 GPa and then they were heated for 2 h at 723 K. The electrical and thermal contacts were made by a silver lacquer mixture (Degussa Leitsilber 200). Static (dc) magnetic susceptibility was measured in two different cooling modes. In the zero-field-cooled (ZFC) mode, the sample was first cooled down in the absence of an external magnetic field and then investigated while heating in a given magnetic field of *H*_dc_ = 1 kOe. Field-cooled (FC) mode usually followed ZFC run when the same magnetic field was set on at high temperatures and measurements were performed with decreasing temperature. Dynamic (ac) magnetic susceptibility was measured at an internal oscillating magnetic field of *H*_ac_ = 3.9 Oe with an internal frequency of *f* = 1 kHz. Both ac and dc susceptibilities were measured within the temperature range of 2–300 K. Magnetization isotherms were measured at 2 K, 10 K, 20 K, 40 K, 60 K and 300 K in applied external fields up to 70 kOe. Magnetic measurements were made using a Quantum Design MPMS-XL-7AC SQUID magnetometer. The effective magnetic moment was determined using the following equation: ${\mu}_{\mathrm{eff}}=\sqrt{\frac{3{k}_{\mathrm{B}}C}{N_{\mathrm{A}}{\mu}_{\mathrm{B}}^2}}\cong 2.828\sqrt{C}$ (Groń et al. 1995; Krok-Kowalski et al. 1997), where *k*_B_ is the Boltzmann constant, *N*_A_ is the Avogadro number, *μ*_B_ is the Bohr magneton and *C* is the molar Curie constant.

## Results and discussion

### XRD, SEM and TEM analysis of Mn^2+^-doped nanomaterials

**Fig. 1:**
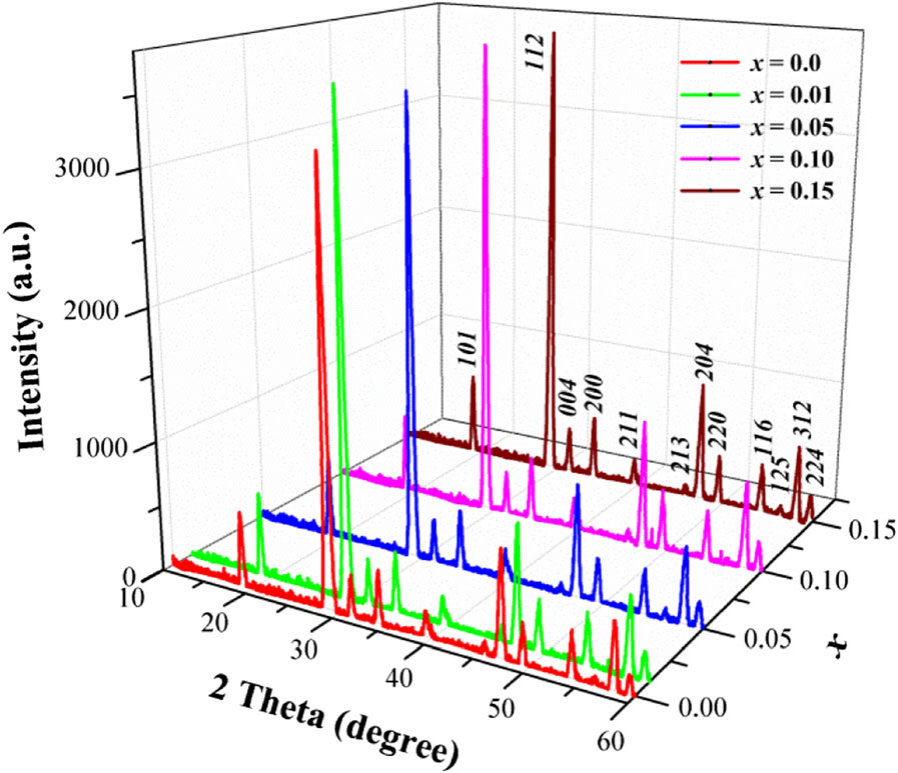
XRD patterns of CaMoO_4_ and Ca_1−*x*_Mn_*x*_MoO_4_ nanomaterials (*x* = 0.01, 0.05, 0.10 and 0.15) heated at 723 K

Figure 1 shows the powder XRD patterns of nanocrystalline CaMoO_4_ and Ca_1−*x*_Mn_*x*_MoO_4_ when *x* = 0.01, 0.05, 0.10 and 0.15 heated at 723 K for 4 h. All powder XRD patterns were indexed based on the JCPDS file number 04-013-6763 for pure CaMoO_4_ with tetragonal scheelite-type structure. No second phases over the entire compositional range were detected indicating the formation of scheelite-type Ca_1−*x*_Mn_*x*_MoO_4_ solid solution. The diffraction patterns of samples when *x* is > 0.15 (not shown here) revealed simultaneously the peaks attributed to Ca_0.85_Mn_0.15_MoO_4_ (the saturated solid solution) as well as the diffraction lines characteristic of manganese molybdate. It means that the maximum solubility of manganese ions in the scheelite-type matrix of CaMoO_4_ is not higher than 15.00 mol% (*x* ≤ 0.15). It was also observed that all diffraction lines of Ca_1−*x*_Mn_*x*_MoO_4_ solid solution shift significantly to a higher 2*θ* angle with increasing manganese ions content due to the substitution of Ca^2+^ (1.12 Å, CN = 8) ions by much smaller Mn^2+^ (0.96 Å, CN = 8) ones (Shannon 1976).

**Fig. 2:**
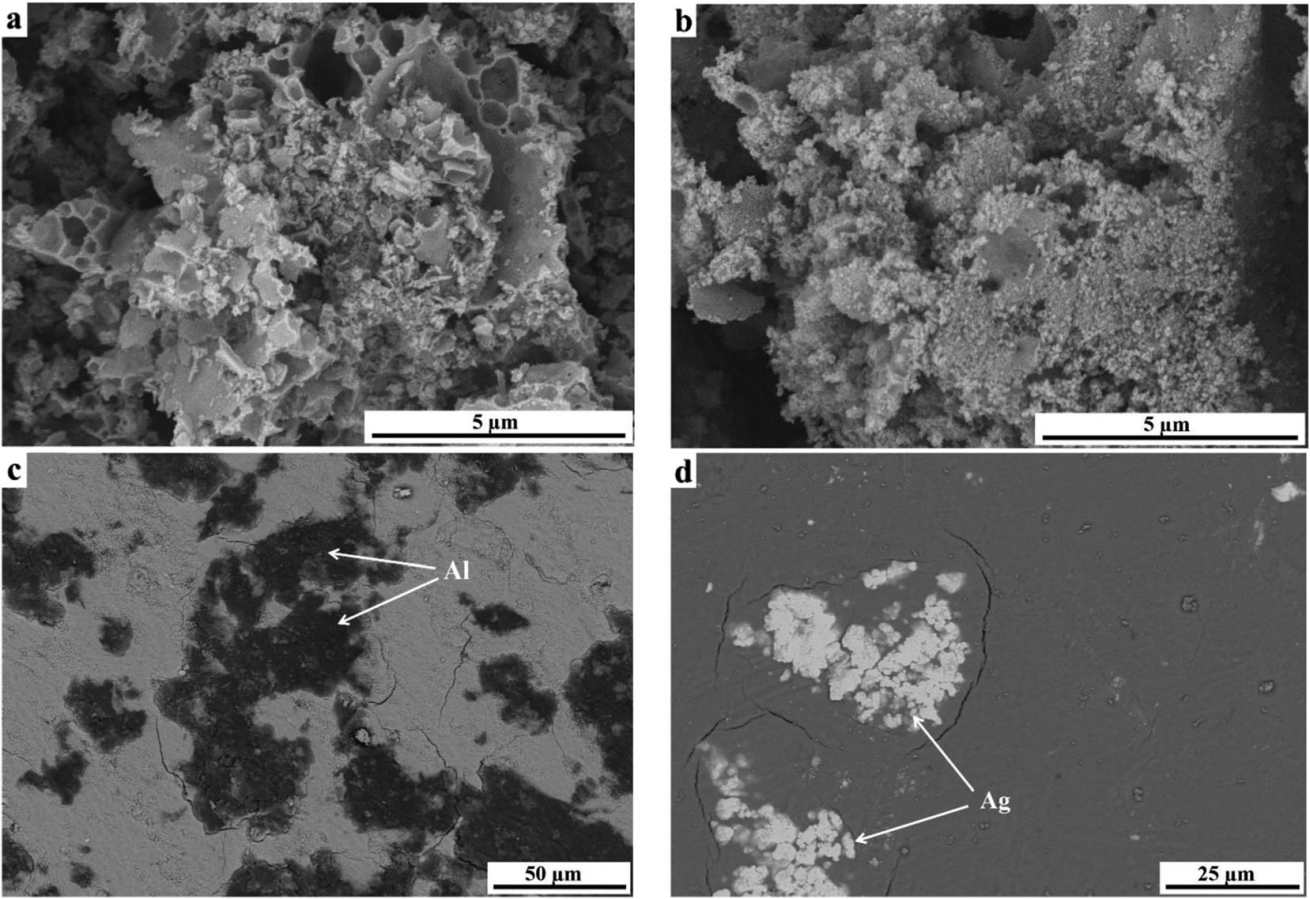
SEM micrographs of CaMoO_4_ (**a**) and Ca_1−*x*_Mn_*x*_MoO_4_ for *x* = 0.05 (**b**) sintered at 723 K. BSE micrographs of co-fired samples with 30 mass% Al (**c**) and 30 mass% Ag (**d**)

**Fig. 3:**
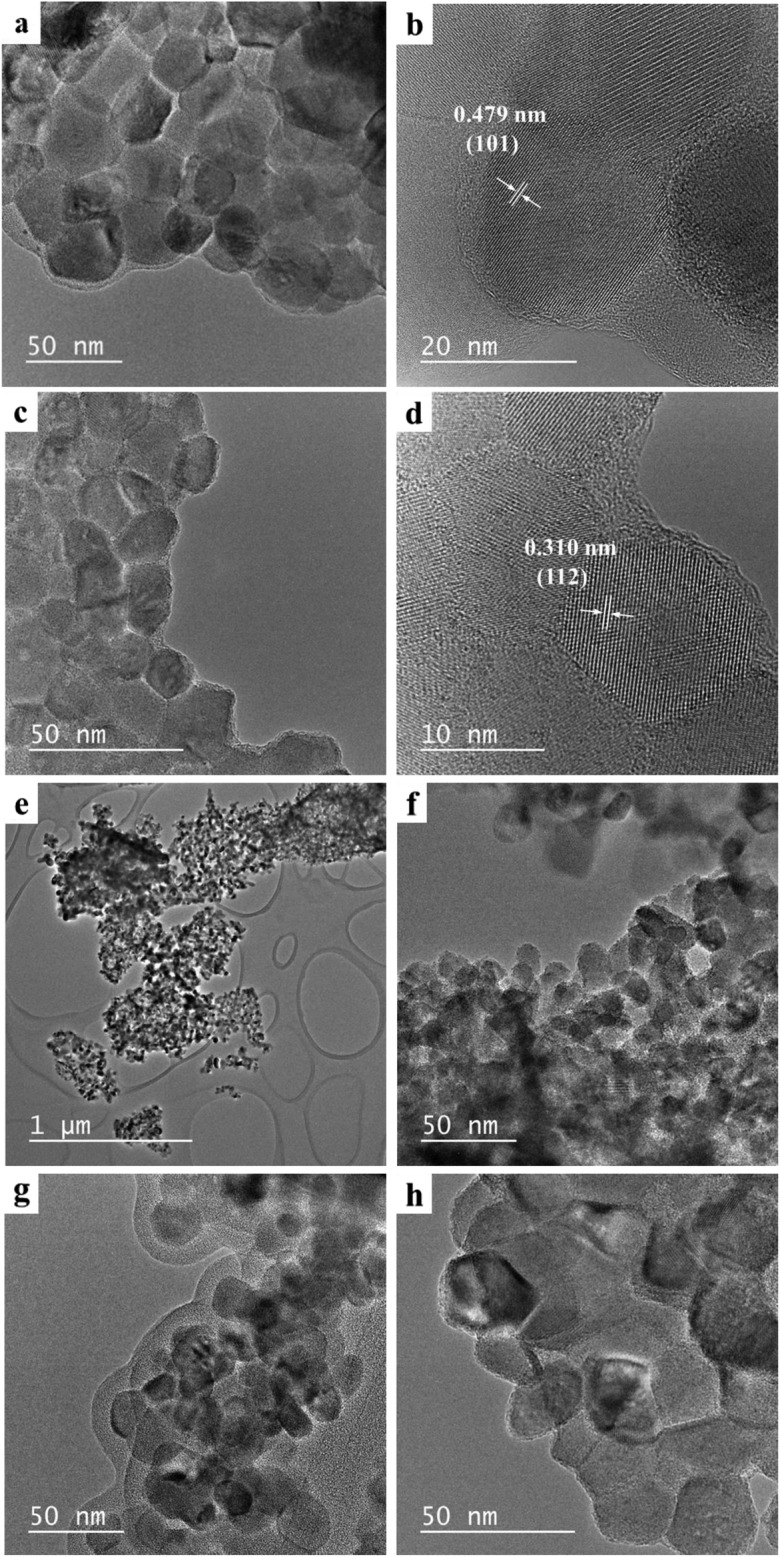
TEM and HRTEM images of CaMoO_4_ (**a**, **b**) and Ca_1−*x*_Mn_*x*_MoO_4_ for *x* = 0.01 (**c**, **d**), *x* = 0.50 (**e**, **f**), *x* = 0.10 (**g**) and *x* = 0.15 (**h**)

**Fig. 4:**
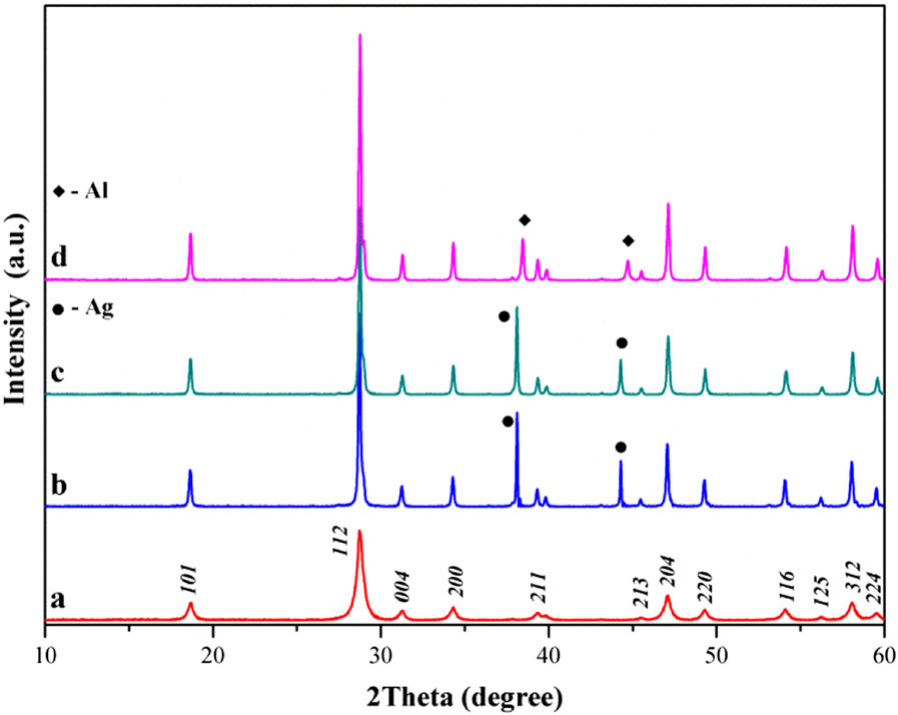
XRD patterns of Ca_1−*x*_Mn_*x*_MoO_4_ (*x* = 0.05) sintered at 473 K (a), CaMoO_4_ co-fired with 30 mass% Ag at 873 K for 4 h (b) and Ca_1−*x*_Mn_*x*_MoO_4_ (*x* = 0.05) co-fired with 30 mass% Ag (c) as well as 30 mass% Al (d) at 873 K for 4 h

The SEM and TEM measurements were performed on as-prepared samples to characterize their morphology, microstructure, particle size and chemical composition. The SEM images are displayed in Fig. 2a–d. The sample of pure matrix (Fig. 2a) is composed of irregular shape of grains differing in their size significantly. Smaller grains are partially agglomerated and form larger aggregates. In contrast, manganese-doped sample (*x* = 0.05, Fig. 2b) contains spherical and much smaller grains only partially agglomerated in bigger clusters. EDS analysis was used to confirm the chemical composition and purity of CaMoO_4_ and manganese-doped nanopowders. EDS analysis (not shown here) of the Mn^2+^-doped sample (*x* = 0.05) heated at 473 K indicated the presence of five elements, i.e. Ca, Mn, Mo, O and C. It means that this temperature is too low for combustion synthesis and the sample was contaminated by amorphous carbon. The presence of carbon has not been confirmed in XRD studies. The powder XRD pattern of the above-mentioned sample consisted of broad diffraction lines which could be associated only to scheelite-type lattice (Fig. 4a). EDS analysis of the Mn-doped sample heated at higher temperature, i.e. 723 K (not presented here also) reveals that the only elements existed were Ca, Mn, Mo and O. No peaks of any impurities were detected, suggesting the high purity of nanocrystalline Mn^2+^-doped calcium molybdate. It was also found that all detected chemical elements are evenly distributed throughout the whole area, revealing a uniform proposed chemical composition of this sample. Figure 3a–h show TEM and HRTEM images of CaMoO_4_ and Mn^2+^-doped nanomaterials. As can be seen from the TEM images, all samples are composed of uniform and oval grains. The grain size of pure matrix and doped nanomaterials varies between ~ 20 and ~ 50 nm, and a significant change in the grain size with increasing Mn^2+^ content in the samples was not observed (Fig. 3a, c, e–h). The HRTEM images presented in Fig. 3b, d give more details on the morphology and suggest that the nanoparticles with grain sizes of about 10–20 nm are highly crystallized. The high-magnification TEM image of the part in the plane of figure shows the measured interlayer spacing of 0.479 nm and 0.310 nm that corresponds to the (101) and (112) crystallographic planes of scheelite-type structure (JCPDS no. 04-013-6763), respectively (Fig. 3b, d).

To assess the chemical compatibility of new nanomaterials with Al and Ag internal electrodes, 30 mass% of these powder metals was mixed with CaMoO_4_ and Ca_1−*x*_Mn_*x*_MoO_4_ when *x* = 0.05 and co-fired at 873 K for 2 h. Backscattered electron (BSE) images and XRD patterns of the co-fired samples are shown in Figs. 2c, d and 4b–d, respectively. Only diffraction lines of scheelite-type lattice and Al (JCPDS no. 04-016-2981) or Ag (JCPDS no. 04-016-1389) were observed in powder XRD patterns of co-fired samples, implying that both CaMoO_4_ and Ca_1−*x*_Mn_*x*_MoO_4_ (*x* = 0.05) nanomaterials did not react with metallic Al and Ag (Fig. 4b–d). Manganese-doped samples and co-fired with Al as well as Ag exhibited two distinct phase grains (Fig. 2c, d). The corresponding EDS analysis shows that the black-coloured grains are metallic Al (Fig. 2c) and the white ones are Ag (Fig. 2d), which is in well agreement with XRD results (Fig. 4c, d).

### Electrical studies

**Fig. 5:**
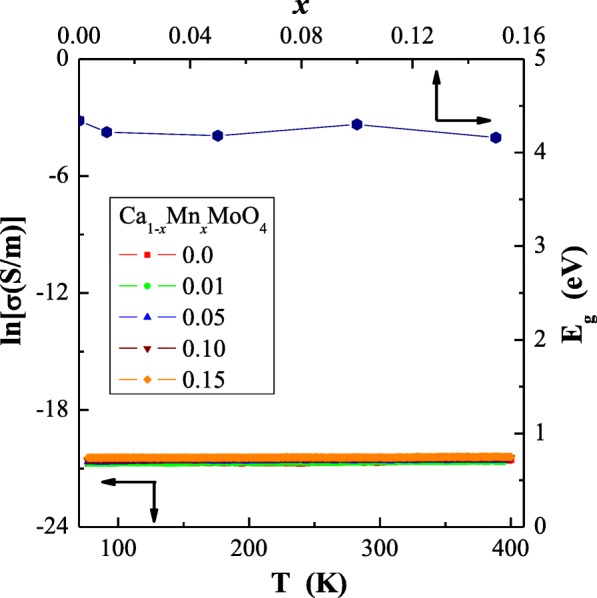
Electrical conductivity (ln*σ*) vs. temperature (*T*) and band energy gap (*E*_g_) vs. *x* parameter for Ca_1−*x*_Mn_*x*_MoO_4_

**Fig. 6:**
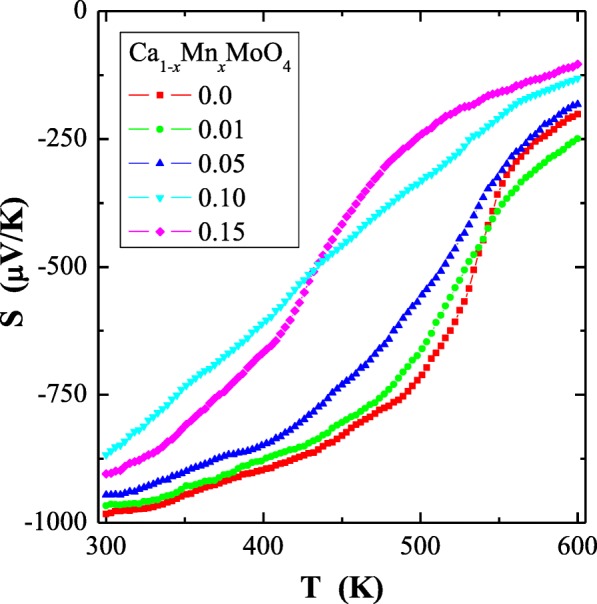
Thermoelectric power (*S*) vs. temperature (*T*) for Ca_1−*x*_Mn_*x*_MoO_4_ (*x* = 0.0, 0.01, 0.05, 0.10 and 0.15)

Results of the electrical measurements of CaMoO_4_ and Ca_1−*x*_Mn_*x*_MoO_4_ (*x* = 0.01, 0.05, 0.10 and 0.15) nanomaterials showed insulating behaviour with small values of the *n*-type electrical conductivity of *σ* ~ 10^−9^ S/m, independent of the manganese ions content (Figs. 5 and 6). This behaviour well correlates with the band gap values (*E*_g_) determined by us earlier (Pawlikowska et al. 2017), and they are displayed in Fig. 5. The above-mentioned values are slightly smaller than 4 eV, and in principle, they do not depend on the content of manganese ions in a sample. No thermal activation of the current carriers was observed. Similar behaviour was found in other materials , i.e. R_2_WO_6_ tungstates (R = Nd, Sm, Eu, Gd, Dy and Ho) (Urbanowicz et al. 2009), CdRE_2_W_2_O_10_ (RE = Y, Pr, Nd, Sm and Gd–Er) (Kukuła et al. 2012; Kukuła et al. 2013) and Cd_1−3*x*_Gd_2x_□_x_MoO_4_ molybdates (where 0 < *x* ≤ 0.2222, and □ denotes cationic vacancies) (Sawicki et al. 2015). The residual electrical conduction of *n*-type in the nanocrystalline molybdates under study seems to be connected with anionic vacancies. Other explanation may be related to the fact that in a state of thermal equilibrium, structural defects (*n*) are always present in the lattice even in the crystal which is ideal in other respects. A necessary condition for free energy minimalization gives *n* ≅ *N*exp(− *E*_V_/*kT*) for *n* ≪ *N*, where *N* is the number of atoms in the crystal and *E*_V_ is the energy required to transfer the atom from the bulk of the crystal on its surface (Kittel 1971). Therefore, we expect deep trap levels localized in the energy gap of 4.2 eV (Pawlikowska et al. 2017) hindering the electron transport.

### Dielectric properties

**Fig. 7:**
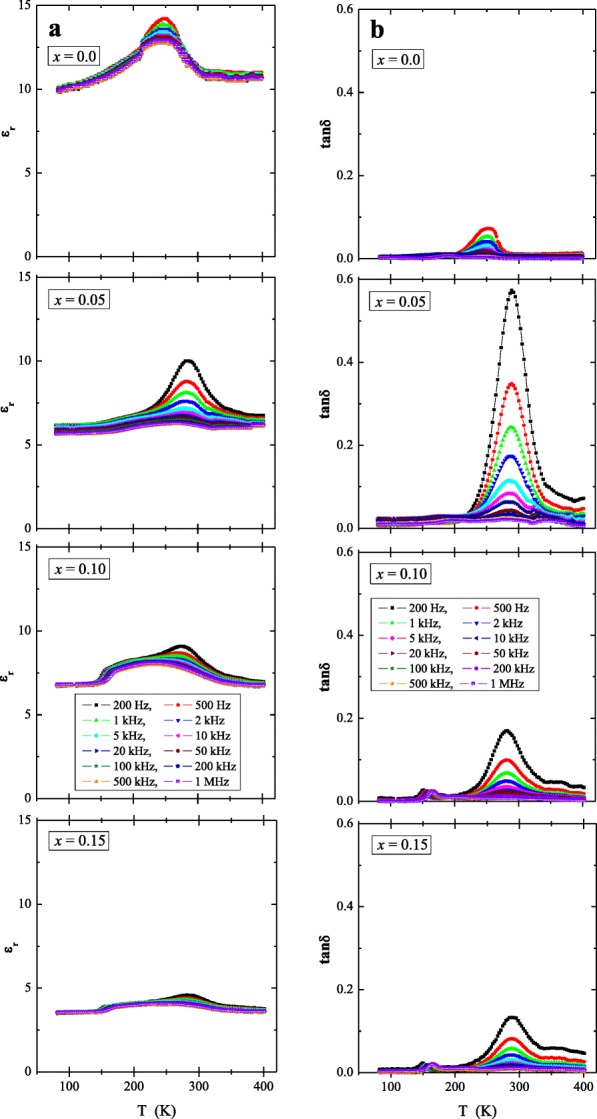
Relative permittivity (*ε*_r_) (**a**) and loss tangent (tan*δ*) (**b**) vs. temperature (*T*) for Ca_1−*x*_Mn_*x*_MoO_4_ (*x* = 0.0, 0.05, 0.10 and 0.15) in the frequency range from 500 to 1 MHz

Studies of broadband dielectric spectroscopy of pure CaMoO_4_ and Ca_1−*x*_Mn_*x*_MoO_4_ (*x* = 0.05, 0.10 and 0.15) nanomaterials displayed in Fig. 7a, b showed low relative dielectric permittivity (*ε*_r_ < 15) decreasing with increasing Mn^2+^ content, its weaker dependence on temperature and frequency except for the broad maximum between 150 and 350 K (Fig. 7a), and the loss tangent (tan*δ*) with the highest value up to 0.6 for *x* = 0.05 (Fig. 7b). For all samples, the dielectric constant and the loss tangent decrease with increasing frequency. The temperature coefficient of dielectric constant (*τ*_*ε*_) between 400 and 100 K at 1 MHz was calculated using the equation shown below (Vidya et al. 2013)

$$ {\uptau}_{\upvarepsilon}=\frac{\upvarepsilon_{400}-{\upvarepsilon}_{100}\ }{300}\cdot \frac{1}{\upvarepsilon_{100}}\cdot {10}^6 $$

The calculated *τ*_*ε*_ values for Ca_1−*x*_Mn_*x*_MoO_4_ are 198 ppm/K (*x* = 0.0), 292 ppm/K (*x* = 0.05), 0 ppm/K (*x* = 0.10) and 114 ppm/K (*x* = 0.15). Generally, Ca_1−*x*_Mn_*x*_MoO_4_ nanomaterials (except *x* = 0.10) have a positive temperature coefficient of dielectric constant whose value changes non-linearly as a Mn^2+^ content was increasing in samples under study. For comparison, microcrystalline MPr_2_W_2_O_10_ (M = Co, Mn) (Kukuła et al. 2012) and M_2_FeV_3_O_11_ (M = Mg, Zn, Pb, Co, and Ni) (Groń et al. 2017) compounds containing 3*d* elements with the unpaired electrons showed both much higher relative permittivity values and loss tangent than the nanomaterials under study. This may mean that small grain size hampers an accumulation of electric charge in each sample. Usually, a majority charge carriers in insulators can be partially recombined in the deep trapping centers (Li et al. 2001) lying under the bottom of the conduction band. Natural source of these traps can be grain boundaries with depletion layers of adjacent grains, as it has been observed for ZnO varistors (Li et al. 2001) and Nb_2_VSbO_10_ ceramics (Groń et al. 2013) as well as some novel copper/cobalt and rare-earth metal tungstates (Groń et al. 2014). However, for the nanomaterials under study, this does not lead to an accumulation of induced charge or to a blocking of a current cross section by boundary phases under the influence of the applied electric external field.

### Magnetic properties

**Fig. 8:**
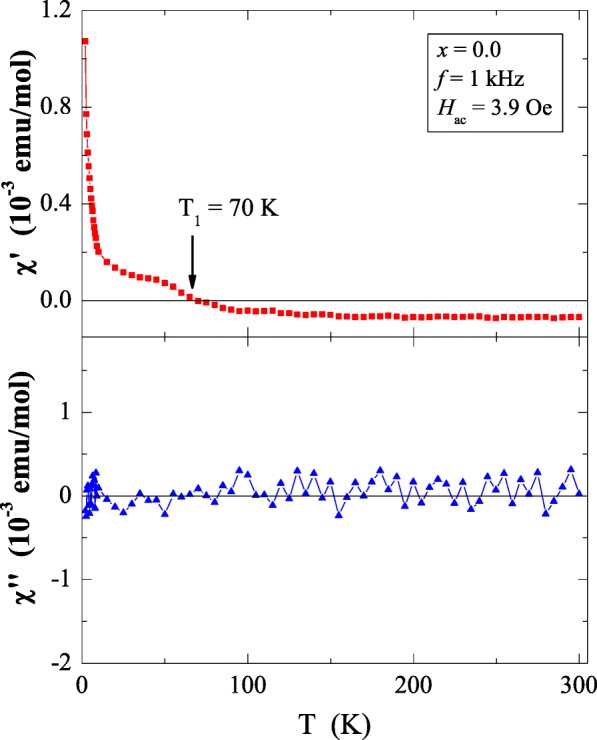
In-phase (*χ*′) and out-of-phase (*χ*″) components of ac magnetic susceptibility vs. temperature (*T*) for CaMoO_4_ recorded at *H*_ac_ = 3.9 Oe with *f* = 1 kHz. The solid (navy) line is the Landé factor fit. The solid (black) line, (*T* − *θ*)/*C*, indicates a Curie-Weiss behaviour

**Fig. 9:**
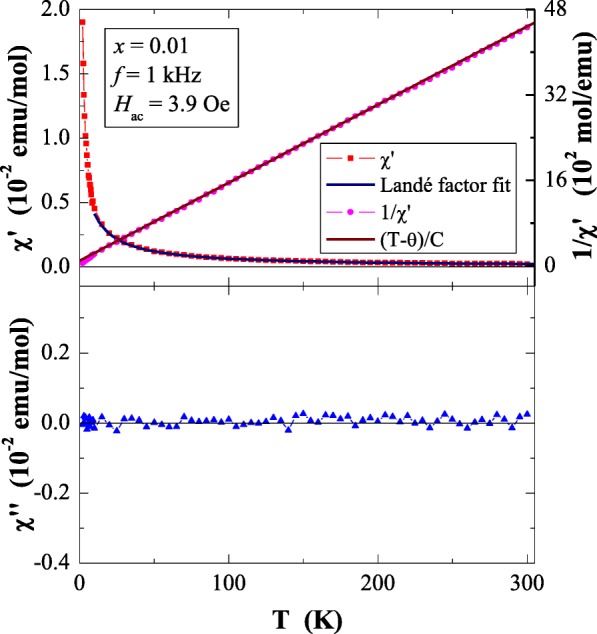
In-phase (*χ*′) and out-of-phase (*χ*″) components of ac magnetic susceptibility vs. temperature (*T*) for Ca_1−*x*_Mn_*x*_MoO_4_ (*x* = 0.01) recorded at *H*_ac_ = 3.9 Oe with *f* = 1 kHz. The solid (navy) line is the Landé factor fit. The solid (black) line, (*T* − *θ*)/*C*, indicates a Curie-Weiss behaviour

**Fig. 10:**
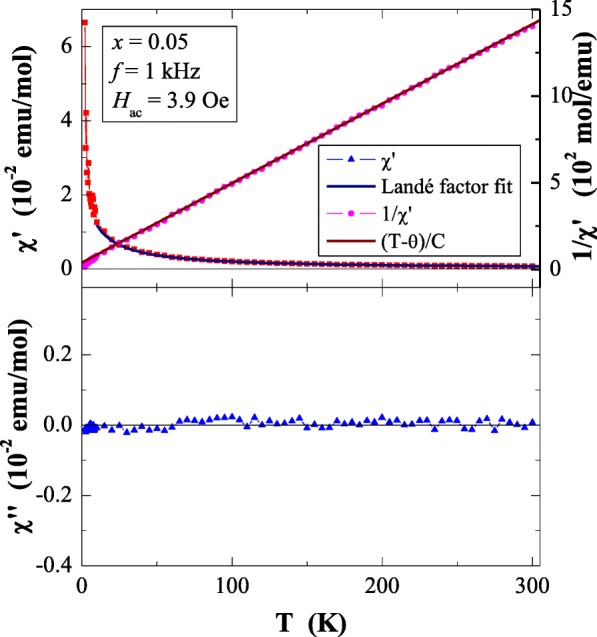
In phase *χ*′ and out of phase *χ*″ components of ac magnetic susceptibility vs. temperature *T* for Ca_1−*x*_Mn_*x*_MoO_4_ (*x* = 0.05) recorded at *H*_ac_ = 3.9 Oe with *f* = 1 kHz. The solid (navy) line is the Landé factor fit. The solid (black) line, (*T* − *θ*)/*C*, indicates a Curie-Weiss behaviour

**Fig. 11:**
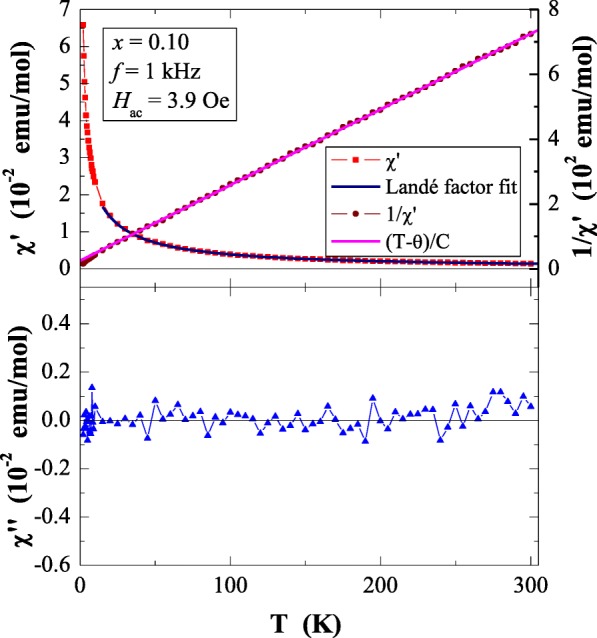
In-phase (*χ*′) and out-of-phase (*χ*″) components of ac magnetic susceptibility vs. temperature (*T*) for Ca_1−*x*_Mn_*x*_MoO_4_ (*x* = 0.10) recorded at *H*_ac_ = 3.9 Oe with *f* = 1 kHz. The solid (navy) line is the Landé factor fit. The solid (black) line, (*T* − *θ*)/*C*, indicates a Curie-Weiss behaviour

**Fig. 12:**
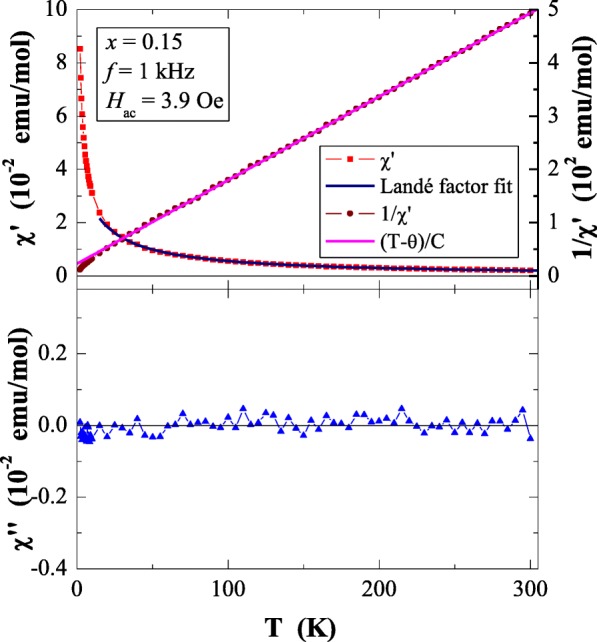
In-phase (*χ*′) and out-of-phase (*χ*″) components of ac magnetic susceptibility vs. temperature (*T*) for Ca_1−*x*_Mn_*x*_MoO_4_ (*x* = 0.15) recorded at *H*_ac_ = 3.9 Oe with *f* = 1 kHz. The solid (navy) line is the Landé factor fit. The solid (black) line, (*T* − *θ*)/*C*, indicates a Curie-Weiss behaviour

**Fig. 13:**
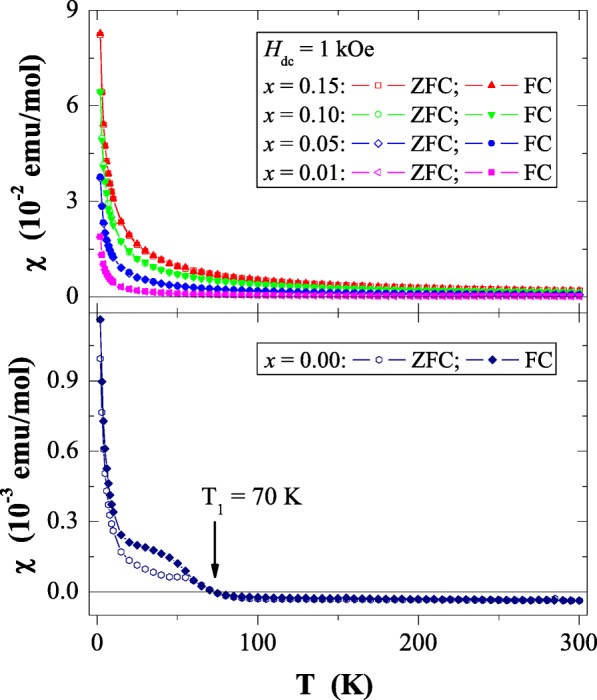
ZFC and FC magnetic susceptibility (*χ*) vs. temperature (*T*) for Ca_1−*x*_Mn_*x*_MoO_4_ (*x* = 0.0, 0.01, 0.05, 0.10 and 0.15) recorded at *H*_dc_ = 1 kOe. *T*_1_ is the paramagnetic-diamagnetic transition for CaMoO_4_

**Fig. 14:**
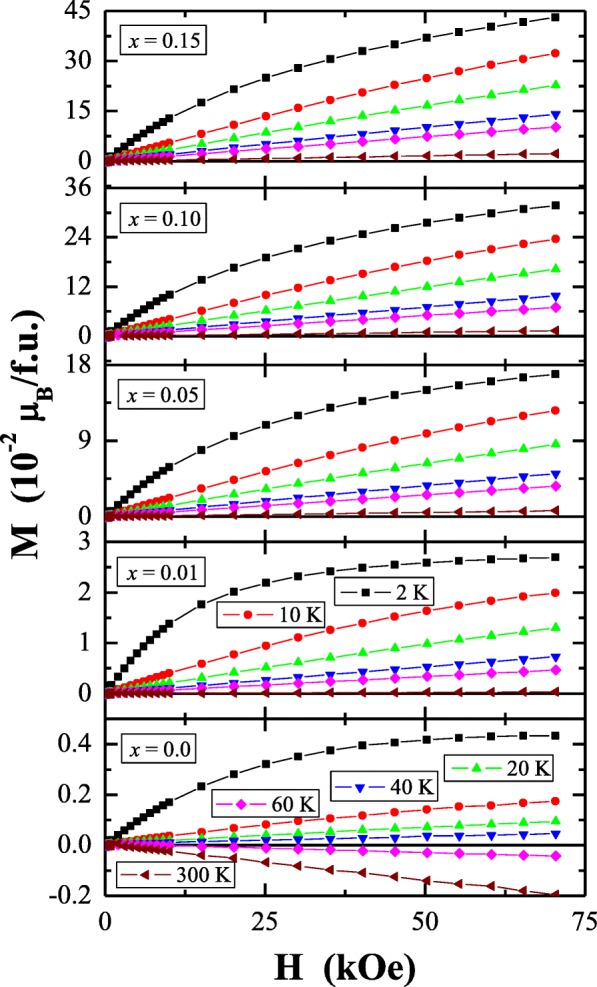
Magnetization (*M*) vs. magnetic field (*H*) at 2 K, 10 K, 20 K, 40 K, 60 K and 300 K for Ca_1−*x*_Mn_*x*_MoO_4_ (*x* = 0.0, 0.01, 0.05, 0.10 and 0.15)

Results of magnetic susceptibility measurements of Ca_1−*x*_Mn_*x*_MoO_4_ (*x* = 0.0, 0.01, 0.05, 0.10 and 0.15) nanomaterials are depicted in Table 1 and in Figs. 8, 9, 10, 11, 12, 13 and 14. All studied molybdates are paramagnetic at low temperatures and have short-range antiferromagnetic interactions visible in the negative values of Curie-Weiss temperature. Oscillating around zero the imaginary component of ac susceptibility (Figs. 8, 9, 10, 11 and 12) as well as no splitting between the ZFC and FC magnetic susceptibilities (Fig. 13) for any phase suggest no long-range interactions and spin frustration within the temperature range of 2–300 K. The effective magnetic moment (*μ*_eff_) is comparable (except for *x* = 0.01) with the effective number of Bohr magnetons (*p*_eff_) for the Mn^2+^ ion with the effective spin of *S* = 5/2, given by the 2[*S*(*S* + 1)]^1/2^ expression (Morrish 1965). This fact can mean that the magnetic moment originates only from a spin. This is also confirmed by the value of Landé factor (*g*) estimated from the Curie constant (Table 1) and obtained by fitting the spectra to a spin Hamiltonian in EPR studies (Dai et al. 2012). Nanocrystalline CaMoO_4_ (Figs. 8 and 13) requires a special attention because it does not have paramagnetic ions, and exhibits residual paramagnetism at low temperatures both on the curve of susceptibility and on the magnetic isotherms. Moreover, when the temperature was increasing, we observed the transition from paramagnetic to diamagnetic, which took place at *T*_1_ = 70 K. Probably, the reason for this behaviour may be an appearance of a small amount of molybdenum ions with unpaired electrons on the 4*d* orbitals. These ions could also have an effect on the magnetic properties of the sample containing the minimum amount of manganese ions (*x* = 0.01) for which a deviation from the spin magnetism (*g* = 2.5 in Table 1) was observed. Results of magnetic moment measurements of molybdates under study are shown in Table 1 and in Fig. 14. Magnetic isotherms do not have hysteresis, coercive field, remanence and saturation. Paradoxically, only pure CaMoO_4_ without paramagnetic ions has saturation at 70 kOe. When manganese content was increasing, the remaining samples were increasingly difficult to magnetize. The reason for this may be magnetocrystalline anisotropy. At room temperature, magnetic isotherms in Fig. 14 show diamagnetism for *x* = 0.0, the balance between diamagnetism and paramagnetism for *x* = 0.01 and paramagnetic behaviour for 0.05 ≤ *x* ≤ 0.15.

**Table 1:** Magnetic parameters of CaMoO_4_ and Ca_1−*x*_Mn_*x*_MoO_4_ nanomaterials

*x*	*C* (emu·K/mol)	*θ* (K)	*μ*_eff_ (*μ*_B_/f.u.)	*p*_eff_	*M*_(2K)_ (*μ*_B_/f.u.)	*g*
0.0	–	–	–	–	4.3·10^−3^	–
0.01	0.0684	− 6.6	0.740	0.592	5.4·10^−2^	2.50
0.05	0.218	− 8.5	1.320	1.324	1.7·10^−1^	1.99
0.10	0.428	− 10.6	1.849	1.871	3.2·10^−1^	1.98
0.15	0.637	− 14.5	2.257	2.291	4.3·10^−1^	1.97

*C* is the Curie constant, *θ* is the Curie-Weiss temperature, *μ*_eff_ is the effective magnetic moment, *p*_eff_ is the effective number of Bohr magnetons, *M* is the magnetization at 2 K and in the magnetic field of 70 kOe and *g* is the Landé factor estimated from the Curie constant

## Conclusions

Nanocrystalline CaMoO_4_ and Mn^2+^-doped calcium molybdates with the formula of Ca_1−*x*_Mn_*x*_MoO_4_ (*x* = 0.01, 0.05, 0.10 and 0.15) were successfully prepared by combustion method. The structural investigations showed the formation of tetragonal, scheelite-type structure (space group *I4*_*1*_*/a*) at 723 K without evidence of any other phases. The electrical measurements revealed that obtained nanomaterials are *n*-type paramagnetic insulators with low relative dielectric permittivity, loss tangent and, in the most Mn^2+^ concentrations, a positive temperature coefficient of dielectric constant at 1 MHz. The chemical compatibilities of Ca_1−*x*_Mn_*x*_MoO_4_ with Al and Ag powders at 873 K make nanomaterials under study suitable for LTCC applications. The magnetic studies showed short-range antiferromagnetic interactions and magnetic contribution coming only from the spin. Pure CaMoO_4_ unexpectedly revealed the residual paramagnetism at low temperatures derived probably from the molybdenum ions having unpaired 4*d* electrons whose effect is a paramagnetic-diamagnetic transition at 70 K. The most interesting conclusion is that reducing grains to nanosizes makes it difficult to accumulate electric charge.
